# Supplementary material for: Aging Disrupts Tissue Homeostasis and Constrains Blastema‐Mediated Regeneration in the Cladonema Medusa
Source: Aging Cell. 2026 Oct 1;25(10):e70727. doi: 10.1111/acel.70727 (PMC13631409; doi:10.1111/acel.70727)
Supplement: Supplementary file 1 [file ACEL-25-e70727-s001.pdf]

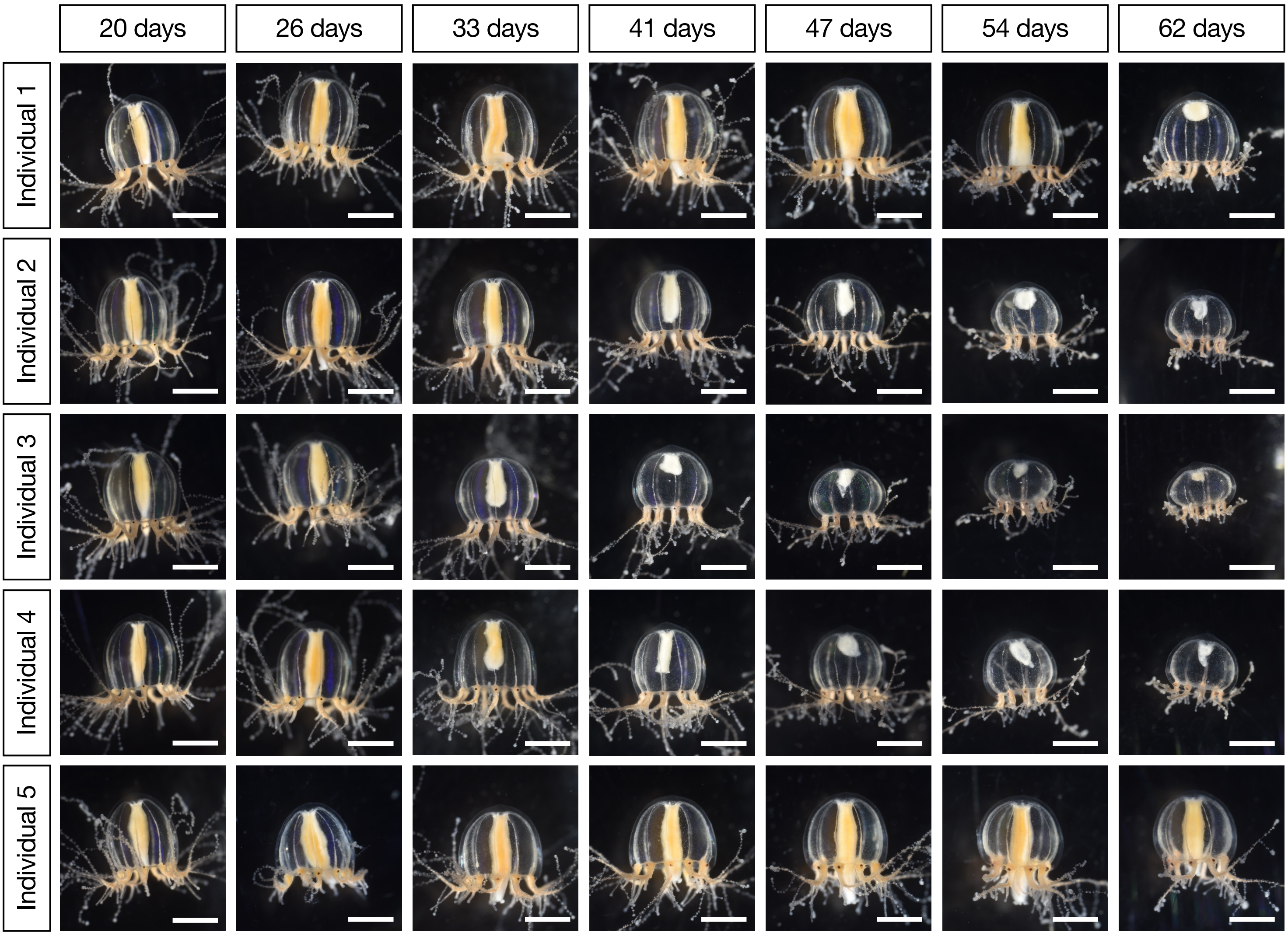

Kanehisa et al.

Figure S1

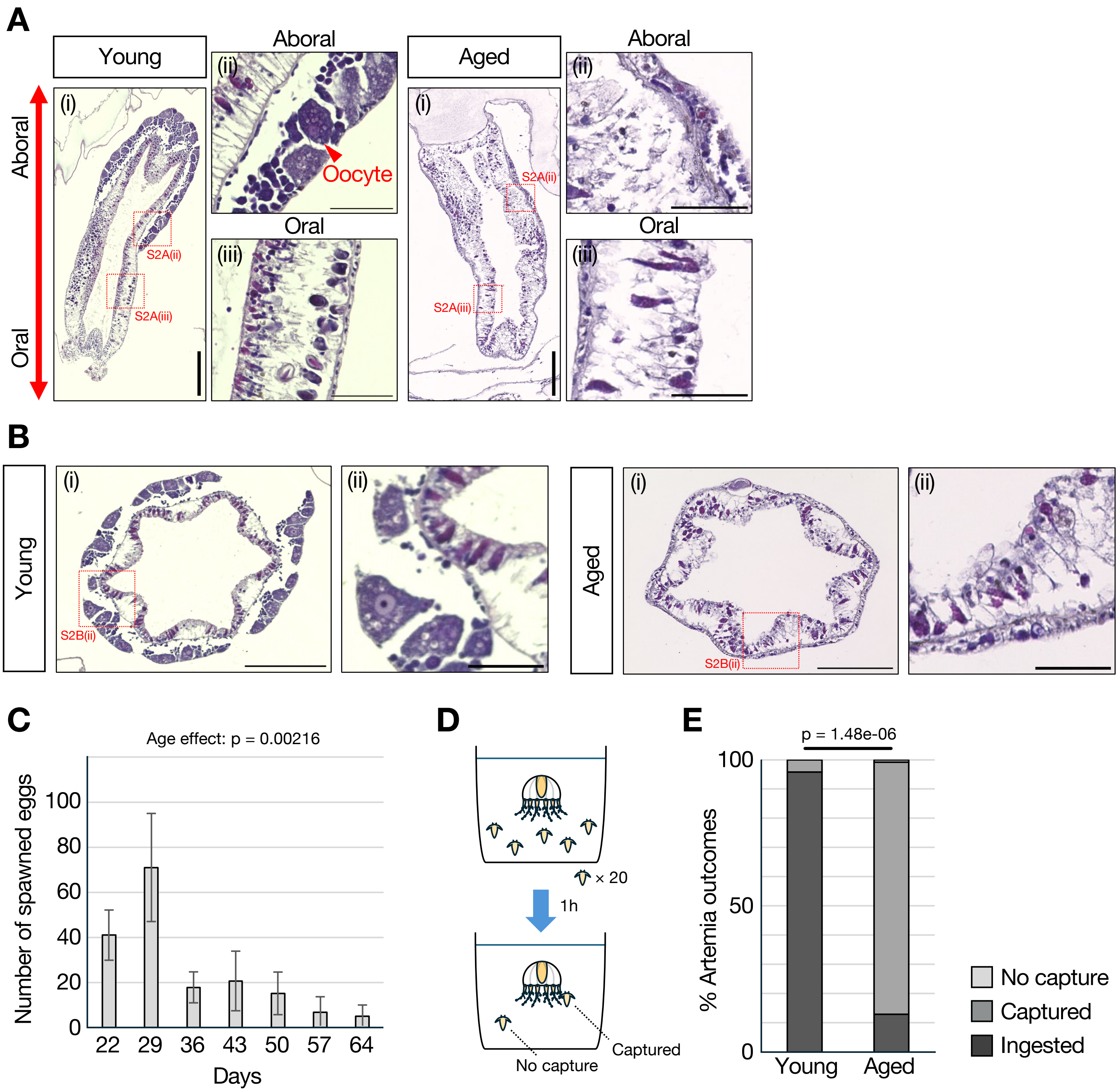

Kanehisa et al.

Figure S2

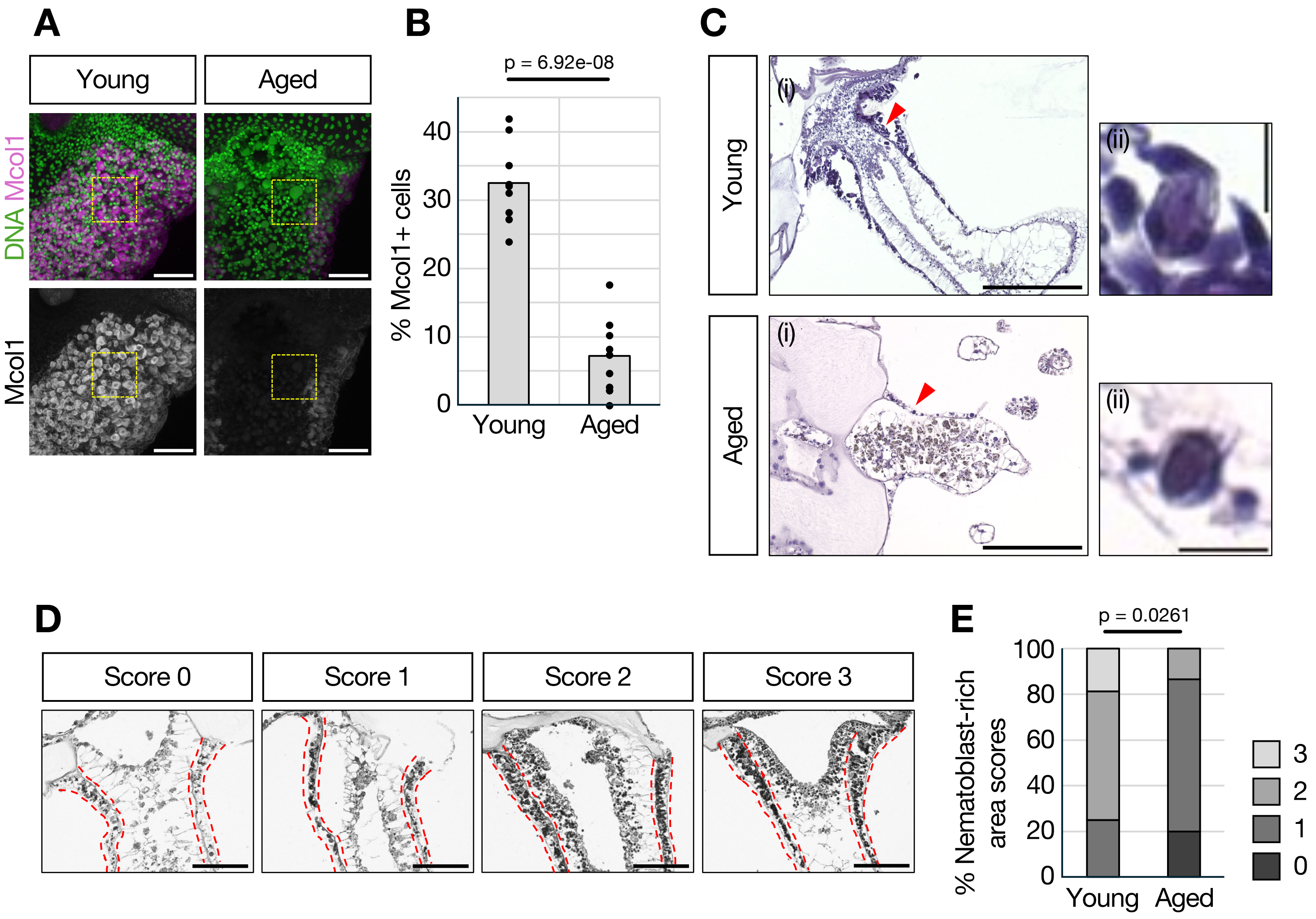
A
Young
Aged
DNA Mcol1
Mcol1
B
p = 6.92e-08
% Mcol1+ cells
40
30
20
10
0
Young
Aged
C
Young
Aged
(i)
(ii)
D
Score 0
Score 1
Score 2
Score 3
E
p = 0.0261
% Nematoblast-rich area scores
100
80
60
40
20
0
Young
Aged
3
2
1
0

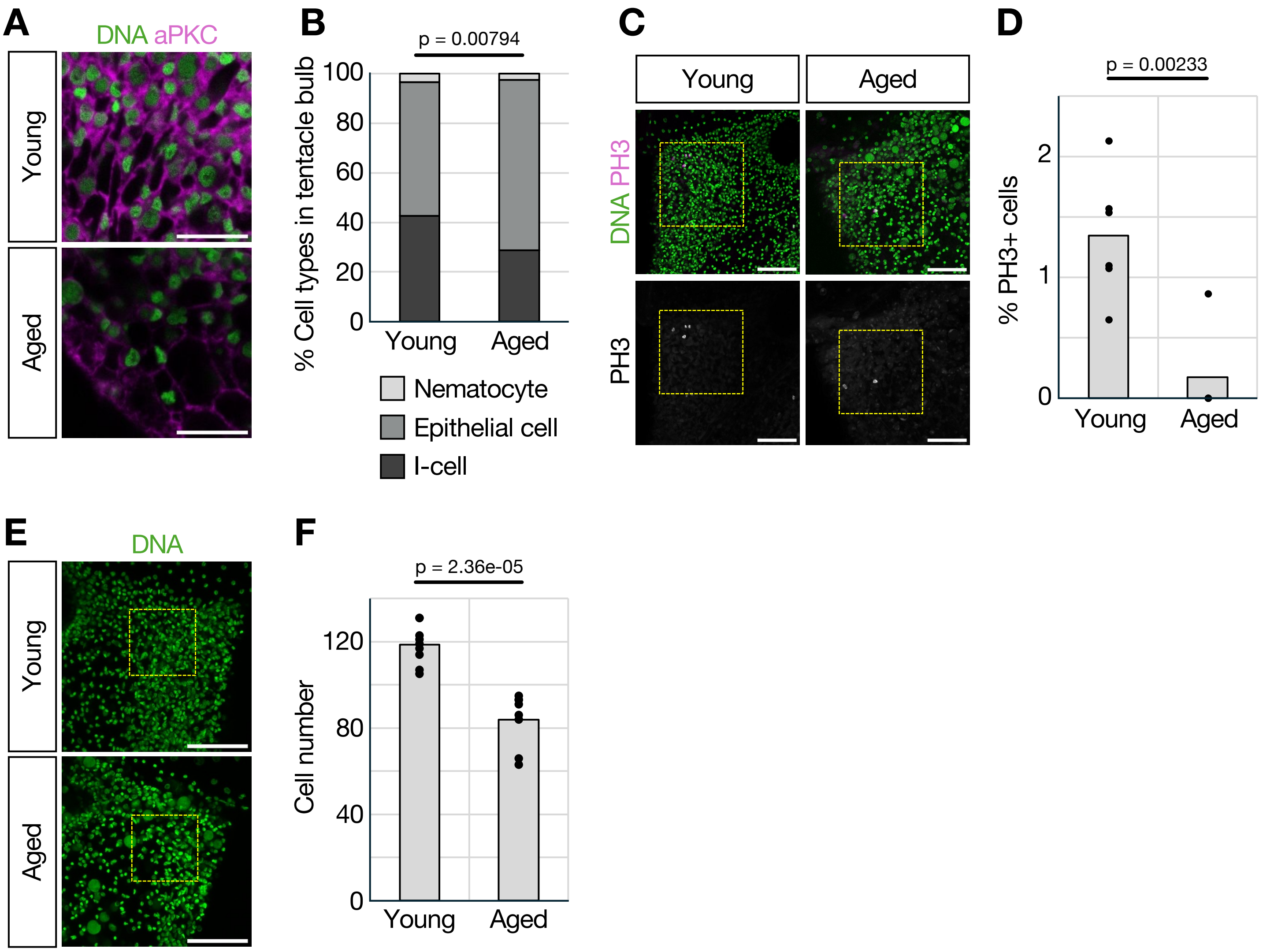

Kanehisa et al.

Figure S4

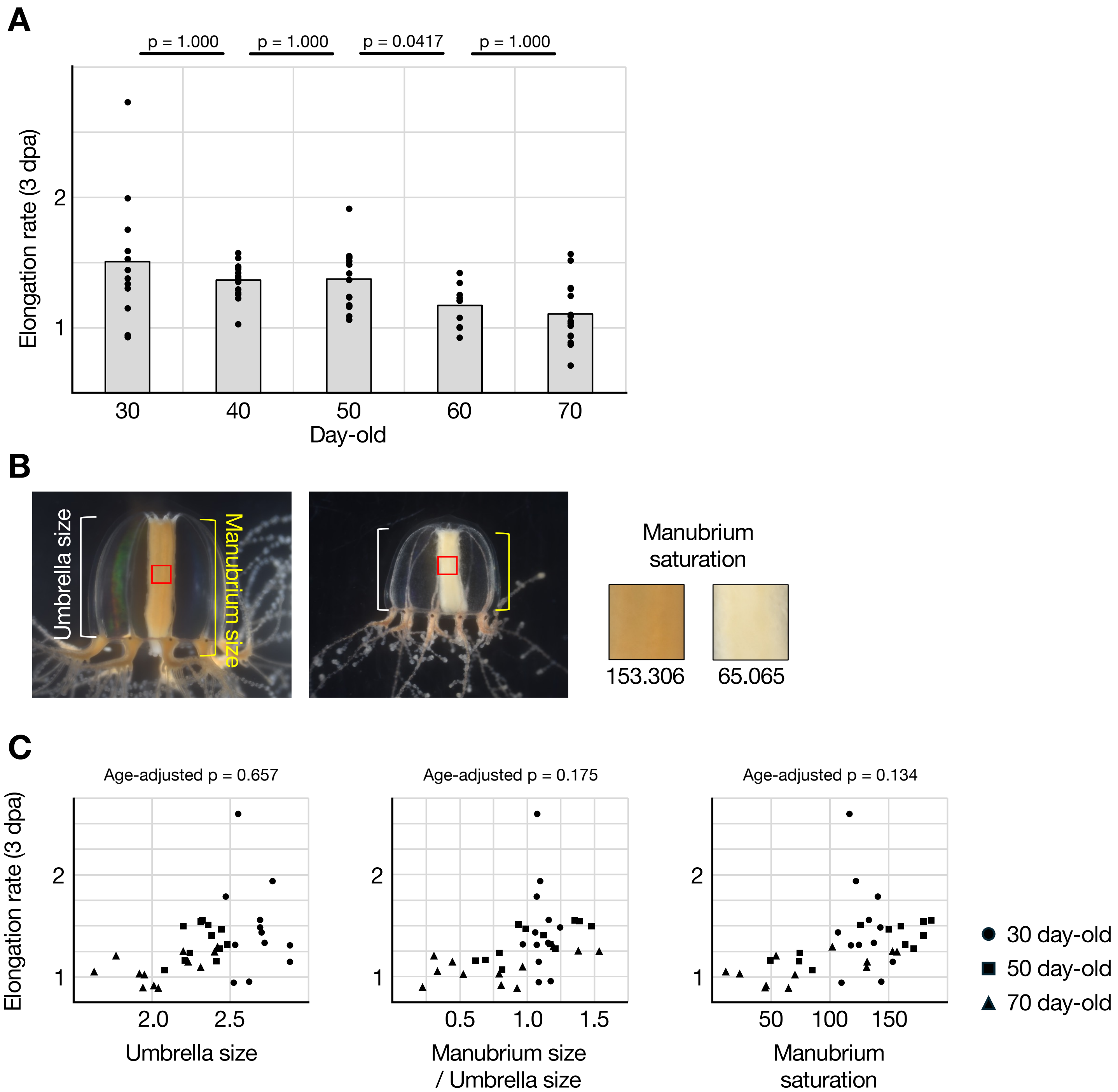

Kanehisa et al.

Figure S5

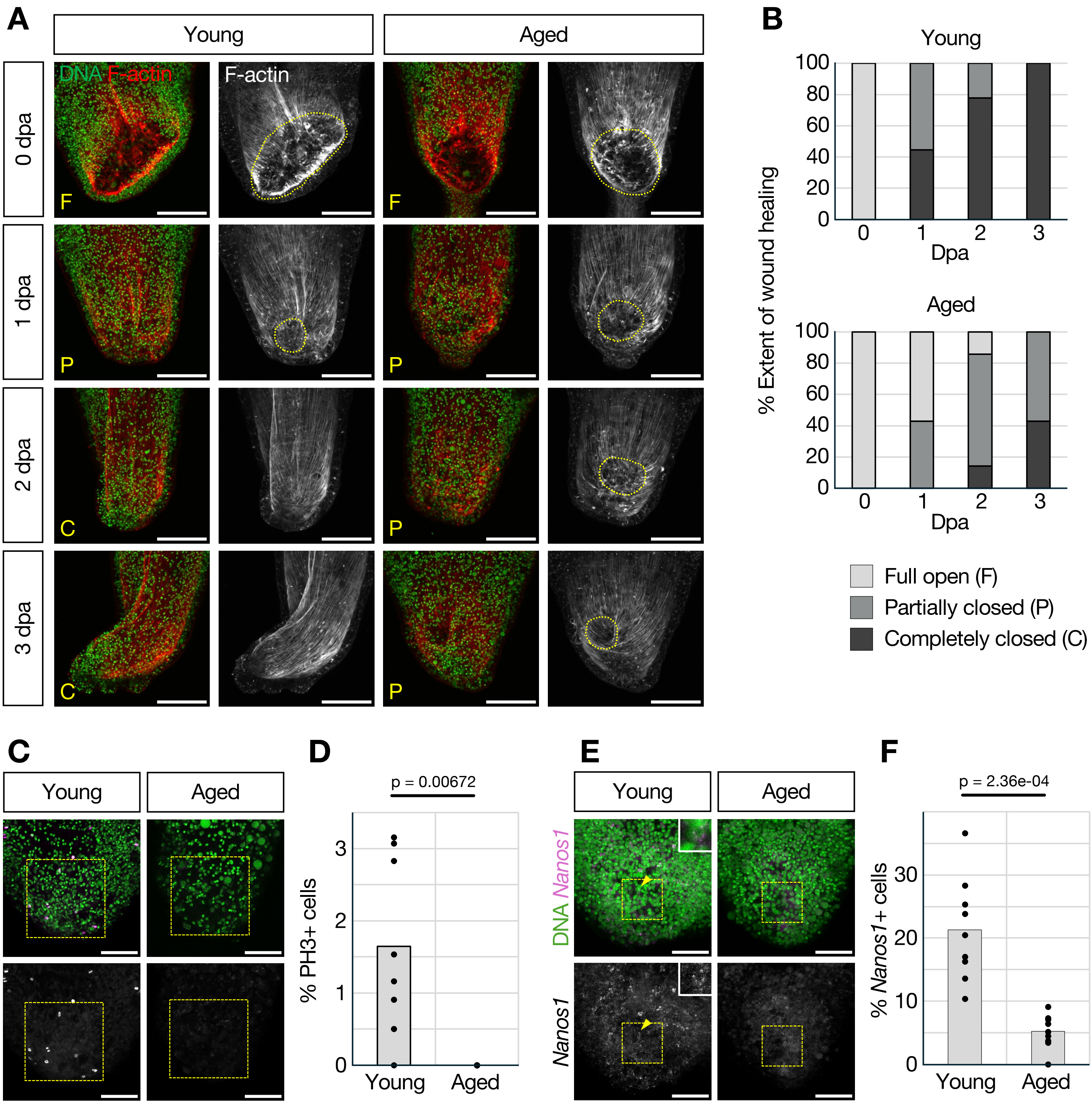

Kanehisa et al.

Figure S6
